# Neurons and a sensory organ in the pedipalps of male spiders reveal that it is not a numb structure

**DOI:** 10.1038/s41598-017-12555-5

**Published:** 2017-09-22

**Authors:** Lenka Sentenská, Carsten H.G. Müller, Stano Pekár, Gabriele Uhl

**Affiliations:** 1 Department of Botany and Zoology, Masaryk University, Brno, Czech Republic; 2 Zoological Institute and Museum, Department of General and Systematic Zoology, University of Greifswald, Greifswald, Germany

## Abstract

The primary function of male copulatory organs is depositing spermatozoa directly into the female reproductive tract. Typical male copulatory organs are sensorily active. This is in contrast to the copulatory organs of male spiders (i.e. palpal bulbi), which have been assumed to lack nerves and muscles until recently. Neurons have been found within the bulbus of the spider *Hickmania troglodytes*, a taxon basal to all Neocribellata. We provide the first evidence for neurons and an internalized multi-sensillar sensory organ in the bulbus of an entelegyne spider (*Philodromus cespitum)*. The sensory organ likely provides mechanical or chemical feedback from the intromitting structure, the embolus. We found further neurons associated with two glands within the bulbus, one of which is likely responsible for sperm extrusion during mating. These findings provide a new framework for studies on reproductive behaviour and sexual selection in spiders.

## Introduction

The primary function of male copulatory organs is depositing spermatozoa directly into the female reproductive tract. The complexity of most animal genitalia and their rapid and divergent evolution strongly suggest that copulatory organs have evolved to such diversity in the context of cryptic female mate choice and sperm competition, and may even function as internal courtship devices^1^. Accordingly, typical male copulatory organs are equipped with a wealth of muscles and nerves, are able to expand and move and are sensorily active. This is in contrast to the copulatory organs of spider males, which are considered unique in that they lack nerves. In spiders, male copulatory organs are separated from the site of sperm production in the opisthosoma. Instead they are shifted to the prosoma, where they are situated on a pair of modified legs, i.e.pedipalps, which are also present in females. The male pedipalp has become highly specialised: sperm are taken up into, stored in and released from the distal part of the pedipalp - the palpal bulbus. The bulbus is connected to a cymbium, a structure homologous to the tarsus of the walking legs. The cymbium is richly innervated and equipped with sensory organs such as setae or slit sensilla. In contrast, the bulbus, which is considered to be derived from hypodermal cells that in females and immatures secrete a claw^2–4^ was found to be free of muscles^5–9^, setae or sensilla^10,11^, and neural tissue^4,11–14^. Copulatory organs of spider males share the evolutionary trend of a rapid divergent evolution found in genitalia of other animals^1,9^. While the bulbus of haplogyne spiders is a rather simple, compact structure, the bulbus of entelegyne spiders evolved into a highly diverse and species specific structure composed of sclerites connected by membranes (i.e. haematodocha).The haematodocha is inflated during the sperm transfer by the haemolymph pressure and it changes the conformation of the sclerites. They are engaged in grasping, bracing, pushing, intromitting, and even damaging the female epigyne, a sclerotized external genital structure^11,15–17^. The sensorily blind copulatory organs of spiders that cannot receive feedback in the mating process have attracted much attention in sexual selection research^18^ and have been used a test case for various hypotheses that attempted to explain diversity of copulatory organs^11^.

Conflicting with the notion that spider palpal bulbi are sensorily blind, behavioural observations have suggested that, for example, male spiders sense whether the tip of their bulbus has entered a sperm droplet during sperm induction or not^19^. Although the various palpal movements accompanying genital coupling are often interpreted as failed intromission attempts that are a consequence of the numb structures having difficulties in achieving the proper alignment with the female structures^20,21^, they may instead represent exploratory behaviour or even copulatory courtship serving to stimulate the female^11,22–24^. Furthermore, it has been recorded that males adjust their behaviour once they reach the epigyne of a previously mated female suggesting that this information is perceived through contact of copulatory organs and suggests sensitivity of the intromitting structure^25,26^.

The discrepancy between morphological and behavioural studies was resolved by a recent study in which it was shown that the simply structured, haplogyne-type bulbus of the cave spider *Hickmania troglodytes* is innervated^27^. *S*everal neurons were found attached to the cuticle, suggesting a proprioreceptive function that may enable the male to gain sensory input during sperm induction and copulation. Furthermore, the epidermal exocrine glands within the bulbus appear connected to the nerve suggesting that the secretory activity might be under neural control^27^. *Hickmania troglodytes* belongs to the species poor family Austrochilidae which is considered a sister group to the two major spider clades Haplogynes and Entelegynes, the so called Neocribellates^28–30^, which together comprise about 90% of all known araneomorph spiders. It remains to be assessed if the neural equipment was lost at the base of the neocribellates or if male copulatory organs of all spiders possess nerves. Here, we provide the starting point for revisiting the supposedly sensorily blind male spider bulbi by scrutinizing the promiscuous entelegyne spider *Philodromus cespitum* (Walckenaer, 1802) (Philodromidae). We investigated the male pedipalp of *P. cespitum* by means of histological, ultrastructural and micro-computer tomographic analyses in the course of a study on the origin of the amorphous material that produces the mating plug which males apply onto the epigyne after mating. We found evidence not only for the presence of neural tissue but also for a multisensillar sensory organ in the pedipalp of *P. cespitum*.

## Results

**Figure 1:**
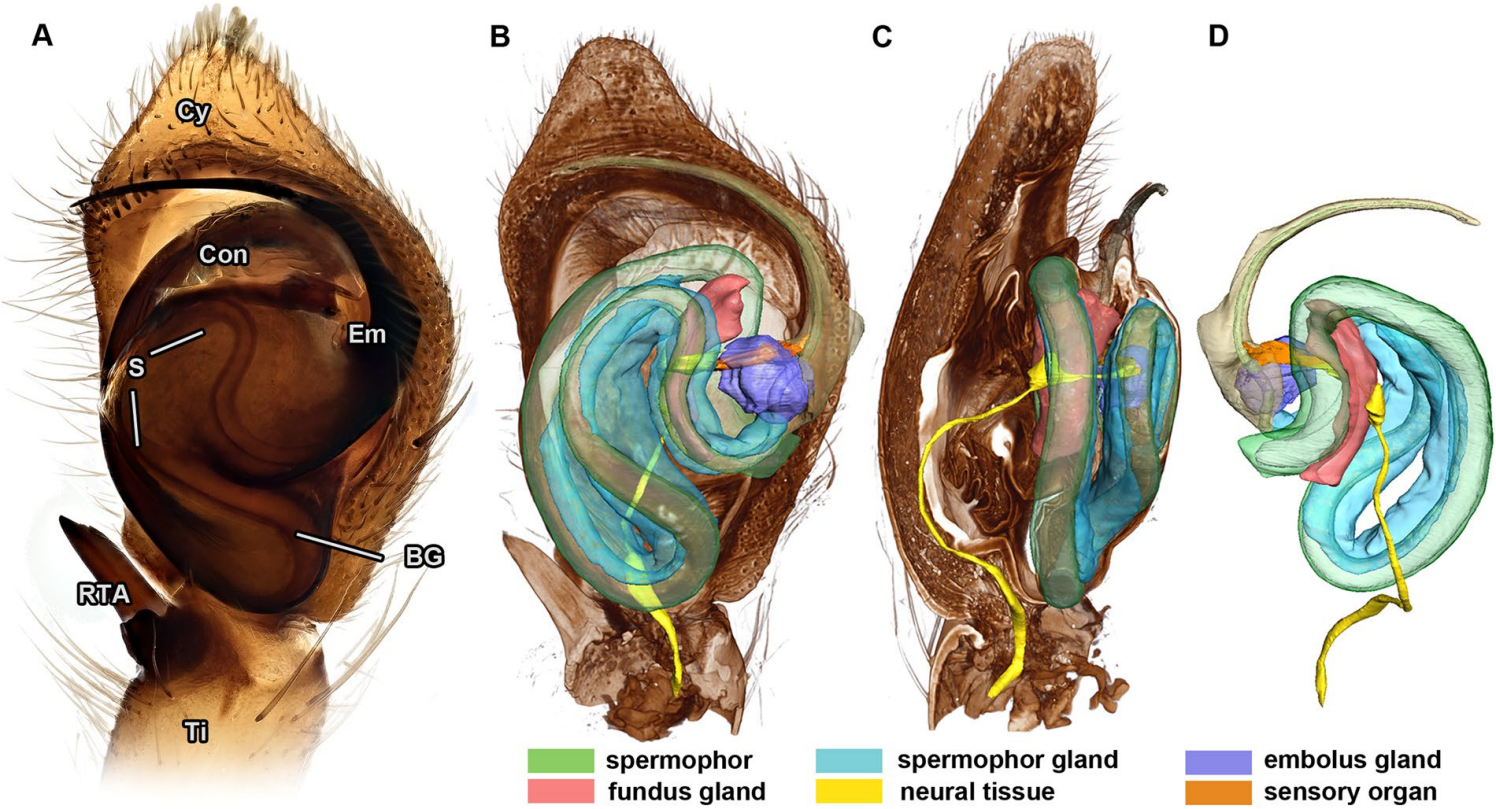
Male pedipalp of *Philodromus cespitum*. (**A**): Ventral aspect (*Visionary Digital BK Plus Lab System)*. (**B–C**): Volume and surface reconstruction of male pedipalp based on *3D X-ray microscopy*, virtual longitudinal sections showing ventral (**B**) and prolateral (**C**) view. (**D**) Dorsal view of delineated focal parts including neural tissue, spermophor, three glandular tissues and embolus. BG – bulbus genitalis, Con – conductor, Cy – cymbium, Em – embolus, RTA – retrolateral apophysis, S – spermophor, Ti – tibia.

The palpal bulbus of *P. cespitum* is an egg shaped structure (Fig. 1A) consisting of two sclerites, tegulum and subtegulum. The bulbus contains the spermophor, a coiled blind tube that functions as an interim storage device for the seminal fluid (Fig. 1B–D). Two sclerites project from the bulbus: the crest-like conductor and the embolus. The embolus has a wide base that narrows distally and bears an opening near its tip. The embolus is the actual intromitting organ; it intakes sperm during sperm induction and releases it into the female epigyne.

**Figure 2:**
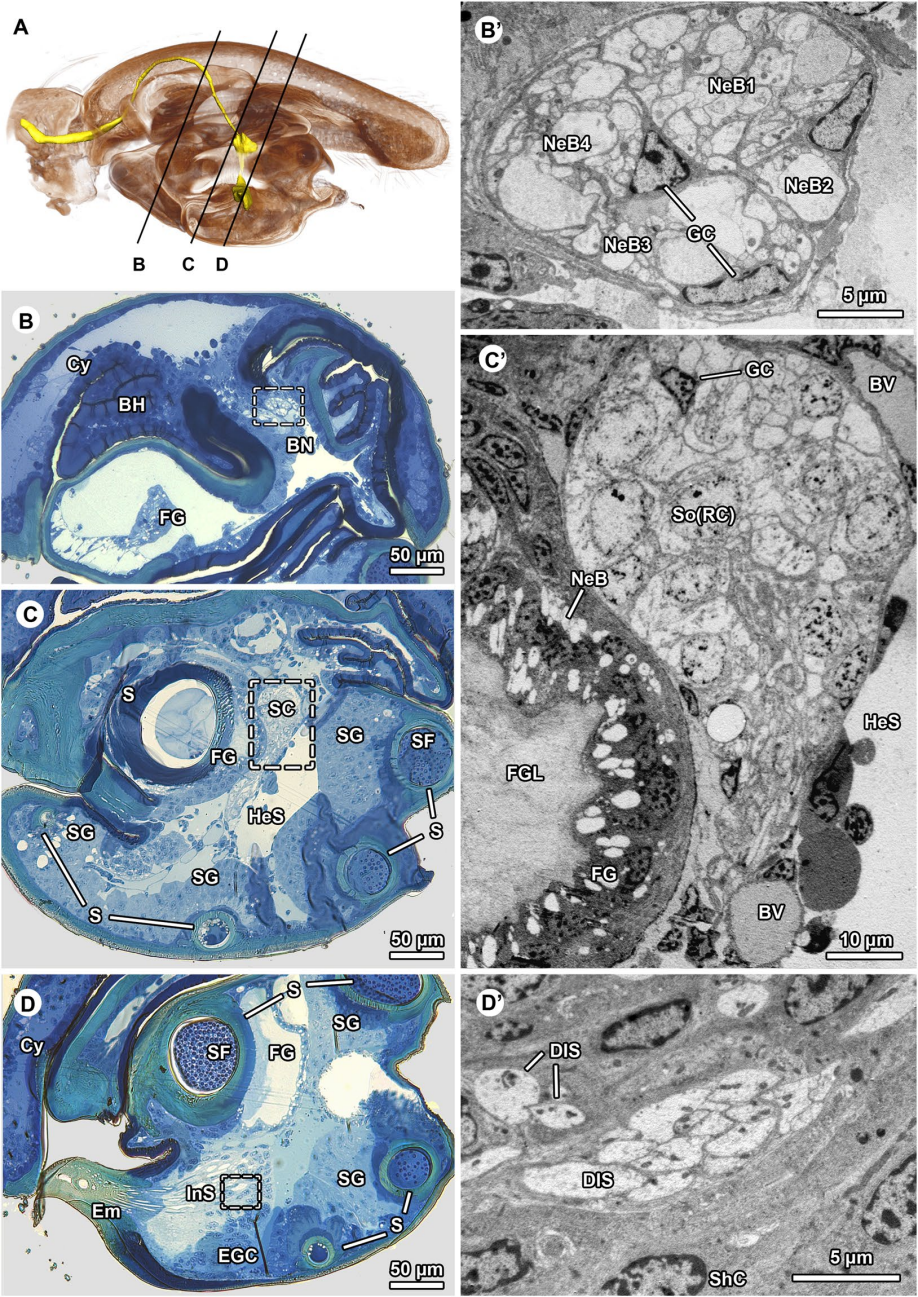
Histological organization and ultrastructure of the palpal bulbus of *Philodromus cespitum*. (**A**) Virtual medio-longitudinal section of bulbus based on *3D X-ray microscopy*. Neural and sensory tissues are highlighted in yellow: bulbus nerve passing through the cymbium and entering the bulbus. Section planes illustrated in (**B**,**C**) and (**D**) are indicated by black lines. Note that further branches of the pedipalp nerve projecting through the cymbium are not highlighted. (**B–D**): Light microscopy; (**B** **–D**): TEM. (**B’–D’**): Sequence of semithin cross-sections of the bulbus showing the location and extension of aggregated internalized sensilla and associated nerve: (**B**) exhibits cross-profile of the bulbus nerve, (**C**) shows the deeply sunk cluster of somata of sensillar receptor cells, while (**D**) highlights distal (dendritic) components of the sensilla. (**B’** **–D’**): Ultrastructural insights from cross-cut bulbus nerve (**B’**) showing neurite bundles in four distinctive compartments as separated by glial cell sheaths. (**C’**): Cell cluster adjoining fundus gland, it contains somata of sensillar receptor cells enwrapped and invaded by glial cells. (**D’**): Proximal aspect of dendritic system of sensilla, pairs or (occasionally) clusters of more than ten dendritic inner segments are visible surrounded by sheath cell somata. BH – basal haematodocha, BN – bulbus nerve, BV – blood vessel, Cy – cymbium, DIS – dendritic inner segment, Em – embolus, EGC – embolus gland cells, FG – fundus gland, FGL – fundus gland lumen, GC – glia cell, HeS – hemolymph space, InS – internalized sensilla, S – spermophor, SC – somata cluster, SF – seminal fluid, SG – spermophor gland, ShC – sheath cell, NeB – neurite bundle, So(RC) – soma of receptive cell.

**Figure 3:**
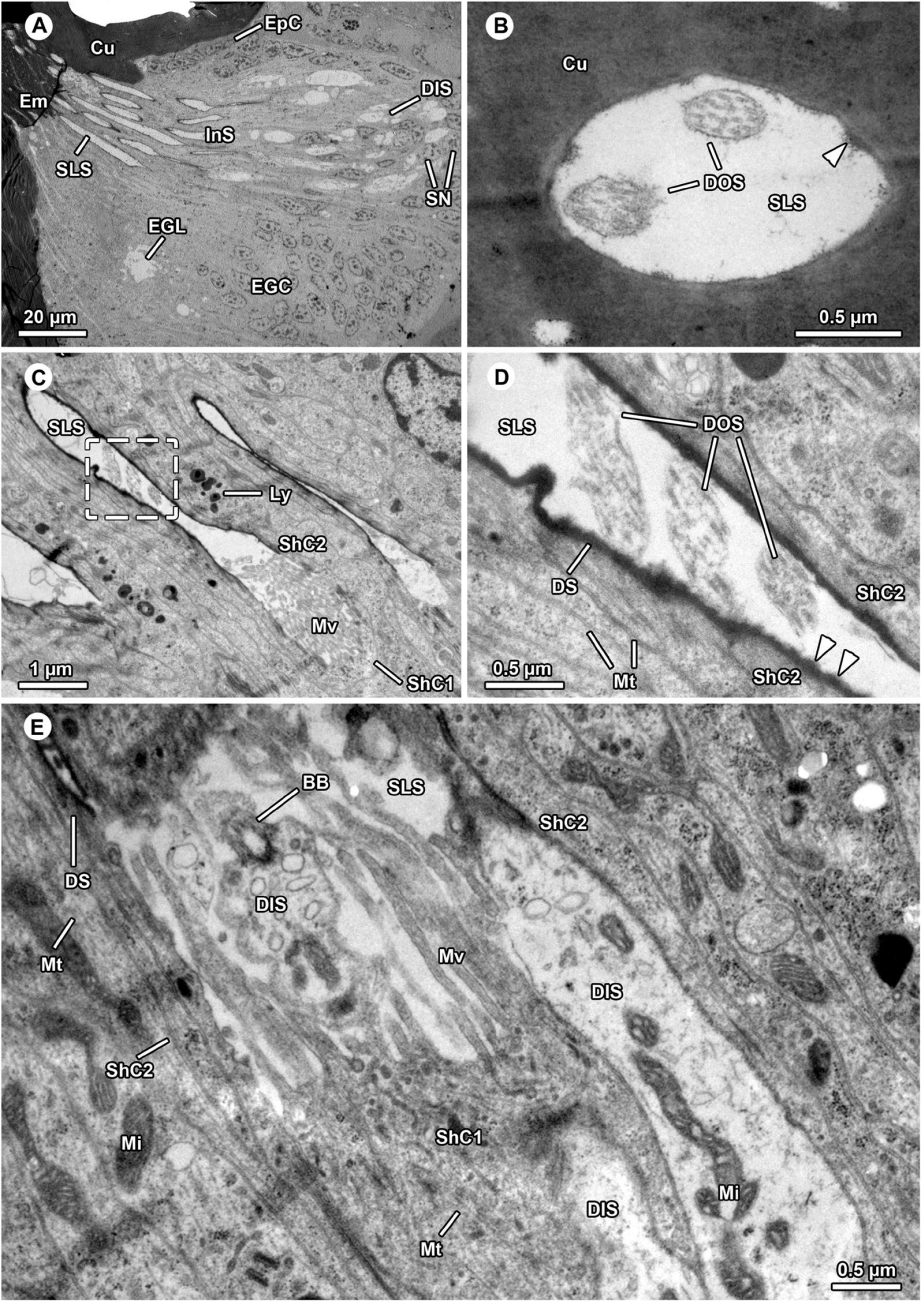
Ultrastructure of aggregated internalized sensilla in the bulbus of *Philodromus cespitum*. (**A**) Overview of distal sensilla components comprising dendrites and sensillum lymph spaces, note that part of embolus gland tissue surrounds the sensilla. (**B**) Most distal, endocuticular part of sensillum penetrating embolus cuticle, electron-lucent sensillum lymph space bears two dendritic outer segments. The aggregation of fibrillous material are potential remains of the dendritic sheath (arrowhead). (**C–D**). In the proximal region the dendritic outer segments project through sensillum lymph space which is lined by highly electron-dense dendritic sheaths (**C**,**D)** close-up of sector boxed in (**C**), sensillum lymph space contains three outer dendritic segments in oblique view. Arrowheads point at knob-like protrusions of the dendritic sheath. (**E**). Transition zone between outer and inner dendritic segments (basal body region), one basal body is visible. The three dendritic inner segments are encompassed by innermost (type-1) sheath cell projecting elongated microvilli into sensillum lymph space. BB – basal body, Cu – cuticle, DIS – dendritic inner segment, DOS – dendritic outer segment, DS – dendritic sheath, Em – embolus, EGC – embolus gland cells, EGL – embolus gland lumen EpC – epidermal cells, InS – internalized sensilla, Ly – lysozomes, Mi – mitochondrium, Mt – microtubuli, Mv – microvilli, SLS – sensillum lymph space, ShC – sheath cell, SN – sheath cell nuclei.

The bulbus is connected to a conical cymbium via a basal haematodocha. Proximally, the cymbium is connected to the tibia. A nerve runs through the tibia into the cymbium where it divides at its base into two distinct nerve bundles. The nerve bundles branch inside the cymbium. One of the branches enters the bulbus (Figs 1C, 2A-B), and is called bulbus nerve henceforth in the text. The bulbus nerve consists of neurite bundles surrounded by glial cells. The glial cells form radial processes that divide the bulbus nerve into three large and one small compartment (fascicles according to Foelix^31^) each carrying a distinct neurite bundle (Fig. 2B’). The bulbus nerve projects inside the bulbus and connects to a cluster of somata adjacent to a gland at the blind end of the spermophor (Fig. 2A,C). Since the blind end is defined as the fundus, we call it fundus gland. The cluster of somata very likely belongs to sensillar receptor cells (Fig. 2C’). The somata contain circular nuclei with little heterochromatin. The cytoplasm around the nuclei is weakly osmiophilic and is equipped with numerous, often elongated or even branched mitochondria of the cristae type, dispersed cisternae of the rough endoplasmatic reticulum (ER), some microtubules, and polymorphic vesicles. The cluster of somata is surrounded and traversed by radiating processes of glial cells (Fig. 2C’) typically associated with neurons. The somata turn into thin distal cytoplasmic processes that project along the ventral margin of the spermophor. These cytoplasmic processes connect to a deeply internalized sense organ situated at the base of the embolus nestled inside the embolus gland (Figs 1D, 2D, 3A).

The sensory organ consists of multiple, tightly adjoined sensilla (Figs 2D, 3A). Each sensillum is innervated by two or three receptor cells, and is surrounded by several sheath cells (Figs 2D’, 3A,B). Based on the terminology that was established for insect sensilla (e.g.^32,33^), the distal process of a sensillar receptor cell soma has to be termed the dendritic inner segment. The dendritic inner segments are assembled in pairs or triads and contain numerous microtubules, mitochondria and small, electron-lucent vesicles (Figs 2D’, 3A,E). Most distally, each dendritic inner segment exhibits a single (sensory) cilium comprising the basal body and axonemal part (ciliary segment, Fig. 3E) as well as the outer dendritic segment - a cilium with microtubules lacking axonemal formation (Fig. 3B–D). Two or three dendritic outer segments pass through the sensillum lymph space that is lined by the thick, highly osmiophilic dendritic sheath (Fig. 3C–E). The rough appearance of the inner surface of the dendritic sheath is caused by fibrillose or knob-like protrusions extending into the sensillum lymph space (Fig. 3D). The dendritic sheath is surrounded and built up by the second sheath cell. The first, basal sheath cell enwraps the dendritic inner segments and builds up and supplies the sensillum lymphs. Along its apex, the first sheath cell emits numerous elongated microvilli protruding into the sensillum lymph space (Fig. 3C,E). Both sheath cell types are characterized by a moderately electron-dense cytoplasm including high abundance of microtubules, mitochondria of the cristae type as well as nuclei with high amounts of heterochromatin, dispersed cisternae of rough and smooth ER, polysomes, Golgi stacks, and polymorphic vesicles with highly osmiophilic, heterogeneous contents resembling lysosomes (Fig. 3C–E). Possibly, there are more than two sheath cells in each sensillum as indicated by the high number of cell layers (6–8) between sensillum lymph spaces (compare Fig. 3C,E). The dendritic outer segments pass through the cuticle of the embolus (Fig. 3A). Here, the sensillum lymph space is lined by remains of the dendritic sheath (Fig. 3B). The course of the dendritic outer segments could not be followed further due to difficulties in sectioning the highly sclerotized embolus.

A further neurite bundle (Fig. 2D’) follows the sensillar dendrites, then bends further ventrally where it connects to the epithelium of the embolus gland situated at the base of the embolus (Fig. 2D). The embolus gland consists of numerous secretory cells arranged in a single but highly prismatic epithelium. The epithelium lines a huge reservoir that narrows anteriorly into a thin canal that runs ventrally to the sensillar apparatus and ends in the embolus canal through which sperm are released.

**Figure 4:**
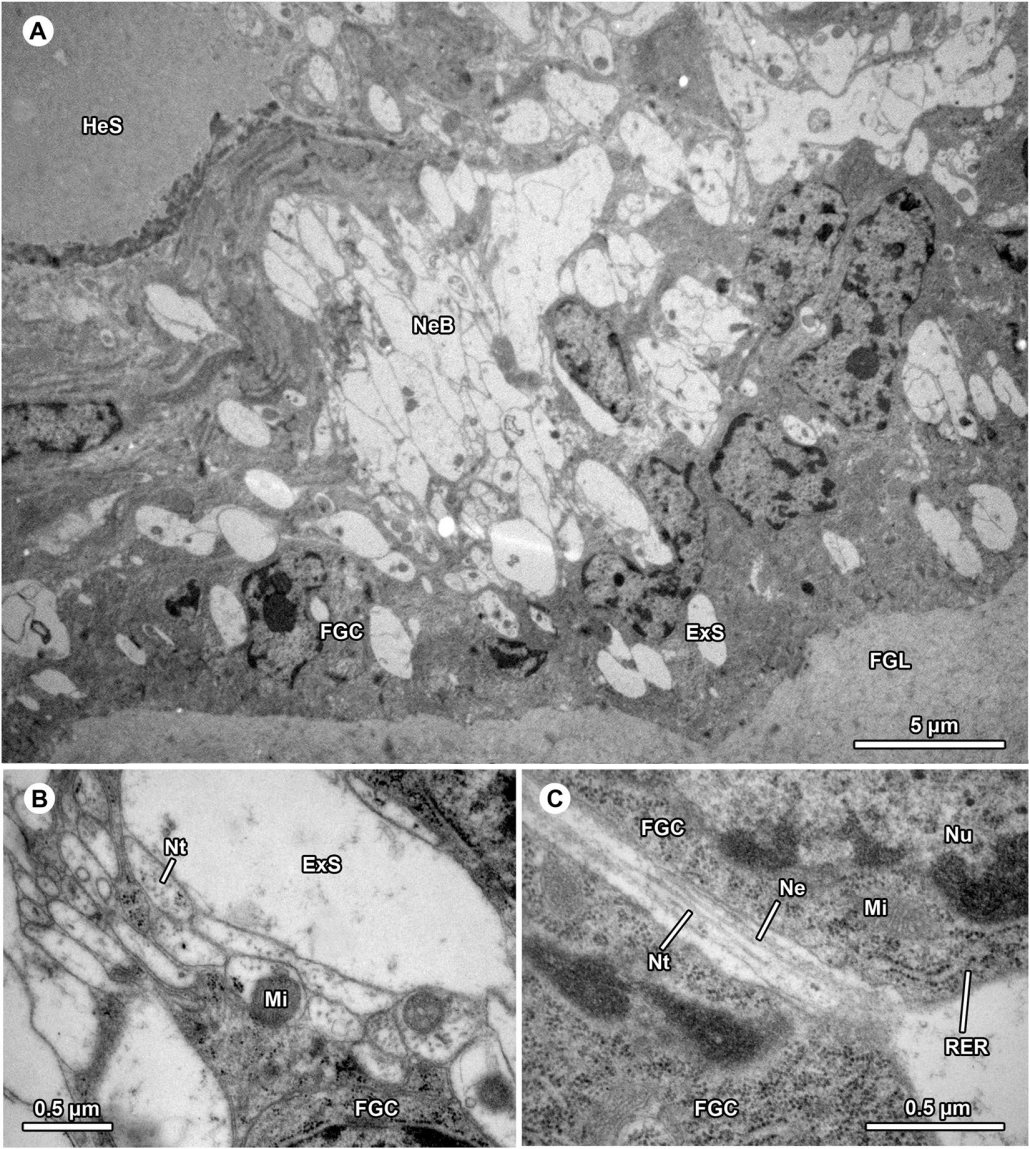
Neural tissue associated with the fundus gland in the bulbus of *Philodromus cespitum*. (**A**) Numerous electron-lucent neuritic inclusions found within the glandular epithelium. (**B**) Neural origin is indicated by presence of neurotubules dispersed in a weakly osmiophilic cytoplasm also containing numerous mitochondria. (**C**) A solitary neuritic process projecting through lateral interspace of two fundus gland cells. DOS – dendritic outer segment, ExS – extracellular space, FGC – fundus gland cells, FGL – fundus gland lumen, HeS – hemolymph space, Mi – mitochondrium, Mt – microtubuli, Ne – neurite, NeB - neurite bundle, Nt – neurotubuli, Nu – nucleus, RER – rough endoplasmatic reticulum.

Another neurite bundle is present in the epithelium of the fundus gland close to the cluster of somata (Figs 2C’, 4A). The fundus gland consists of a thin, single-layered vacuolated epithelium that surrounds a voluminous reservoir filled by an amorphous substance of low electron density (Figs 2C’, 4A). The reservoir connects to the porous wall of the spermophor fundus region. The substance in the spermophor shows similar staining affinities towards toluidine blue (Fig. 2C) suggesting that it originated from the fundus gland. The existence of neurites within the fundus gland is indicated by neurotubules extending through a weakly osmiophilic cytoplasm (Fig. 4B). Single neurites were occasionally detected in between secretory cells (Fig. 4C).

The third gland, the spermophor gland, runs along the spermophor wall between fundus gland and embolus gland for most of its length. It connects to the wall of the spermophor that is porous in the region of contact (Fig. 2 C,D). The spermophor gland does not show any indication of innervation. It comprises many secretory cells that form a thick glandular epithelium rich in rough endoplasmic reticulum and microtubules (not illustrated). The lumen of the spermophor that is surrounded by this gland is filled with sperm and spherical vesicles of high electron density (Fig. 2C,D).

Comparison of male pedipalps before and after copulation revealed differences in the distribution of sperm and glandular secretions: in virgin males that had charged their pedipalp with sperm but did not mate yet, a mixture of sperm and circular vesicles fills three quarters of the spermophor lumen, whereas the substance of the fundus gland fills the distal portion of the spermophor, the fundus, and the voluminous reservoir. In pedipalps fixed right after copulation, the substance of the fundus gland fills two thirds of the spermophor lumen, and the apically remaining lumen contains sperm and vesicles. The other two glands (embolus gland and spermophore gland) showed no obvious differences before and after copulation.

## Discussion

Our study demonstrates that the palpal bulbus of an entelegyne spider possesses neural tissue. Moreover, we provide the first evidence of a multisensillar sensory organ. For several decades it has been accepted that male spider genitalia are sensorily blind due to the lack of neurons in the part of the copulatory organ that is involved in sperm transfer. This notion was built on numerous histological studies^2,9,12–14^. The apparent lack of sensory feedback during genital coupling has been considered to be compensated by the evolution of a number of sclerites that interlock with structures of the female copulatory organ as is typical for most spider species^11^. Recently, however, neural tissue was found in the simple bulbus of the austrochilid cave spider *Hickmania troglodytes* ^27^ which gave the starting point for revisiting the neurobiological properties of the bulbus in a representative of the majority of spiders, the Neocribellates. Interestingly, the arrangement of the nerve and neurons in *H. troglodytes* ^27^ and *P. cespitum* is nearly identical although the bulbi differ considerably between these two species. The neural tissue in the bulbus of *P. cespitum* also consists of a nerve proceeding from the cymbium and a somata cluster inside the bulbus. In both species, the neural tissue reaches the base of the embolus and seems to be tightly associated with glands as the neurites penetrate their tissue. The striking similarity in neural anatomy of two distantly related taxa may suggest that the presence and arrangement of the neural tissue represents a ground pattern for all Araneomorphae and possibly for all spiders.

The nerve that runs in the bulbus of *P. cespitum* is clearly distinguishable from other tissues when using the combination of methods applied in our study. This may explain why the studies mentioned above failed to find neural tissue in bulbi of other neocribellate spider species. Depending on the quality of the chemical fixation process and magnification, the presence of neurons may have been obscured. Especially in paraffin and semi-thin sectioning, which were applied in most of these studies, the neural tissue does not conspicuously stain with the common staining protocols. Also, in specimens only fixed in ethanol the neural tissue does not seem to be preserved well enough to be distinguishable, as was the case for the congener *Philodromus dispar* ^9^. Therefore, we predict that nerves may be found in the palpal bulbi in the whole order Araneae. Whether the nerves have a sensory or motor function or both needs to be assessed in follow up studies. Our results suggest that both directions are conceivable, since the bulbus nerve contains several neurite bundles (Fig. 2B’). The fact that we found a senory organ requires that the input perceived by this organ is transfered and processed to an integrating unit. Consequently, some of the neurite bundles are likely sensory. Since the neurites connect to at least two of the three glands found inside the bulbus, a further motor function of some of the bundles is plausible. These nerves may be involved in the release of secretions and consequently in the expulsion of sperm during mating, a process that likewise is not understood at present. In the following, we first discuss our data on the sensory and motor aspect of the neural equipment of the *P. cespitum* bulbus.

The sensory organ of *P. cespitum* is situated close to the base of the embolus, the intromitting structure of the bulbus. The organ is composed of aggregated and internalized sensilla. The cuticle of the embolus is penetrated by the outer dendritic segments of the sensilla, however, we could not trace their full course in the cuticle. We tentatively suggest that this sensory organ is chemo and mechanoreceptive. We base our hypothesis on ultrastructural details (see below), the connection with the various bulbus glands, and the position of the sense organ in the embolus. In *H. trogylodytes*, neurites were likewise found attached to the cuticle near to the embolus base. As a result of a stress and strain finite-element modelling analysis a proprioreceptive function was posultated^27^. However, our ultrastructural data reveal that the sensilla of the bulbus of *P. cespitum* resemble in some respects the internal set up of the tarsal organ, a chemoreceptor found on the tarsus of the walking legs as well as on the pedipalp of various spiders (e.g.^34–36^). Shared (ultra-)structures are: (1) the tight, palisade-like packing of the numerous, deeply internalized sensillar units, (2) the number of receptor cells and dendritic outer segments emitting from them (usually 3, rarely two), and (3) the elongated and narrowed sensillum lymph space, which is (4) accompanied by a thick dendritic sheath along its entire length. Due to difficulties in sectioning the most apical region of the sensilla, we do not know the full course of the dendrite outer segments. If the bulbus sense organ were homologous to the tarsal organ, the sensilla should terminate in small, cone-shaped, and tip-pored protuberances on the cuticle or in a depression that connects to the outside through a minute pore^36,37^. When scrutinizing the embolus wall under the SEM, we could not detect a porous structure. Thus our study tentatively suggest that the internalized sensilla in the bulbus function either as pheromone receptors^37^ or as combined thermo- and hygroreceptors^38^ as was previously suggested for the tarsal organ or, alternatively, as a proprioreceptor. Our study also documents two neurite bundles that make contact and branch within the glandular epithelia associated with the embolus and fundus. We failed to depict synapses, probably because the fixative does not infiltrate easily into the bulbus. However, there is a reason to assume that these two neurite bundles (see Figs 2D’ and 4A) include axons that innervate and thus regulate the activity of the embolus and fundus gland. Our arguments for assuming that these neurite bundles carry efferents and would therefore have to be interpreted as branches of the bulbus nerve are as follows. First, the bulbus nerve is made up of four distinct compartments of unequal size, so called fascicles^31^. In insects, such nerve compartments generally carry axons of strictly either sensory or motor neurons when distant from the ganglia^38,39^. Accordingly, we assume that the fascicles that are small in diameter contain efferents coming from interneurons located in the brain, each innervating a particular gland, whereas the largest fascicles of the bulbus nerve contains afferents that come from the sensory organ.

The neural equipment of the spider bulbus does not only impact on our perception of the possibilities and constraints that occur during the mating process in spiders. The finding that neurites enter the fundus and embolus glands may considerably help to advance our knowlegde on the mechanism by which sperm is released during mating from the interim sperm storage organ, the spermophor. It has been assumed that sperm expulsion is achieved through increasing internal hemolymph pressure applied on the bulbus by more proximal muscles or by means of a secretion from a gland attached to the spermophor^12,13,40^. Particularly, the spermophor gland was suspected to discharge its product through the porous wall into the lumen of the spermophor during mating^2,13,41^. Our histological examinations of *P. cespitum* bulbus reveal that the reservoir of the fundus gland is filled by a material identical to the one present in the lumen of the spermophor. In virgin males, large amounts of the material are deposited in the glandular reservoir and a small portion can be found in the fundus region of the spermophor. After copulation, however, the gland appears shrunken and the material fills more than half of the spermophor lumen. Our data strongly suggest that the fundus gland plays a crucial role in sperm extrusion and that the process is triggered by a neural stimulus. Since the mating process in spiders generally entails changes in pedipalp and bulbus conformation by internal pressure changes, the pressure pump and the neural stimulus may be coordinated for successful sperm transfer. Lamoral^13^ already reflected on why sperm is only released during mating after embolus insertion despite the fact that the pressure pump is active much earlier in the mating sequence, e.g. for external alignment with the female genitalia. He suspected that the release of a secretion for sperm transfer from the gland is triggered by neurohormones produced in more proximal parts of the male pedipalp, once the pedipalp achieved the right position. However, since sperm transfer often occurs very rapidly, a neurohormonal activation is unlikely^14^. Our study provides a tentative answer to this question since the fundus gland seems to be directly innervated by neurites projecting through the bulbus nerve. The sperm expulsion may be triggered when the sensory organ sends information about the correct positioning of the pedipalp during mating. This afferent transmission may cause the fundus gland to release the substance from its reservoir into the spermophor lumen to flush out the seminal fluid stored therein.

Before sperm release during mating, spider males have to charge their pedipalps. They transfer sperm from the production site (testes in opisthosoma) onto a sperm web and from there into the spermophor of their copulatory organs. Sperm uptake has to be repeated when the spermophor is emptied through copulation. It has been proposed that males take up sperm into the spermophor through resorption of material that fills the spermophor lumen before the bulbi are charged. Thereby the sperm mass is sucked into the spermophor^10,13,42^. We presume that the material we found in the fundus gland and spermophor fundus in virgin males of *P. cespitum* fills the entire spermophor before sperm induction and that it is resorbed by the fundus gland during the process of sperm uptake. Apart from the fundus gland, there are two more glands in the palpal bulbus of *P. cespitum*, the spermophor gland and the embolus gland. The spermophor gland seems to release a secretion into the spermophor before mating in many spider species (Uhl, unpublished) which may explain why there were no obvious differences between the spermophor glands of virgin and mated males in our study. Consequently, it may be involved in sperm uptake. The embolus gland may push the sperm mass out of the spermophor, and into the copulatory duct of the female which might be essential if the copulatory duct is long and the embolus cannot penetrate all the way to the spermathecae inside the female. Additionally, since *P. cespitum* males produce a mating plug by which males can hinder females from remating with rival males (Sentenská, Pekár & Uhl, unpublished) as was shown for other spiders^42–44^, one (or both) of these glands might be involved in producing the mating plug material.

In conclusion, our finding of a neural tissue and sensory organ in the pedipalp of an entelegyne spider together with the previous finding on nerves in the pedipalp of a basally branching Austrolichilid spider requires not only revising the common notion that genitalia of spider males are numb structures. The finding also calls for a reanalysis of the origin of the spider bulbus as a derivative of the tarsal claw^2,45^. This explanation has already been challenged by the observation that traces of a claw as well as of a bulbus occur simultaneously during early development of male pedipalps^11,45^. Overall, our findings offer a new perspective on genitalic sensory feedback and mate assessment in spiders.

## Methods

Subadult males and females of *P. cespitum* (Walckenaer, 1802) were collected with a beating-net in the canopy and understorey of an abandoned fruit orchard within the city boundary of Brno, Czech Republic in April 2015. Spiders were housed individually in a Petri dish (diameter 5 cm, height 1 cm) with a piece of filter paper, which was moistened with few drops of water at 2-day intervals to maintain the required humidity. Spiders were kept at room temperature (approx. 22 °C), at 40% RH, and under a natural LD regime and fed with fruit flies (*Drosophila melanogaster* Meigen) to satiation at 2-day intervals. A day after the final moult the spiders were sent to Greifswald University, Germany and their pedipalps were fixed in Karnovsky fixation (see below) except for two, which were fixed in 70% alcohol. Three of the virgin males were mated with virgin females (approximately five days after the final moult) and their pedipalps were fixed right after the copulation in Karnovsky solution.

A high definition stacking photograph of the external anatomy of the male pedipalp fixed in 70% alcohol of *P. cespitum* was produced with a Visionary Digital BK Plus Lab System (duninc.com/bk-plus-lab-system.html) equipped with a Canon EOS 6D and a customized Canon EF 100mm f2.8 IS USM professional macro lens at the University of Greifswald. The images were processed with Adobe Photoshop CS6.

For histological and ultrastructural analysis, pedipalps of 10 males were fixed overnight in a fresh, cold fixative solution modified after Karnovsky^46^ containing 2.5% glutaraldehyde, 2.5% paraformaldehyde, 1.5% NaOH and 5% D-glucose, buffered with 0.1 M sodium phosphate buffer adjusted at pH 7.4. After rinsing the pedipalps three times in the buffer solution for 5 min, postfixation in 1% OsO_4_ solution (same buffer) was conducted at room temperature for 4 h followed by dehydration in a graded series of ethanols and embedding in Spurr media (SIGMA-ALDRICH).

Semi-thin (500 nm) and ultrathin (50–65 nm) cross sections were made using a Leica ultramicrotome UC6. The semi-thin sections were stained with 1% toluidine blue in a solution of 1% sodium tetraborate (borax; modified after Richardson^47^), and examined and photographed under an Olympus BX60 connected to a Zeiss MCr digital camera. Ultrathin sections were mounted on Formvar-coated slotgrids (PLANO: model G2500C), stained with uranyl acetate and lead citrate for 4 min each, and then examined under a JEOL JEM-1011 transmission electron microscope operated at 80 kV. Images were taken with an Olympus Mega View III digital camera using iTEM software.

For non-invasive X-ray microscopy (XRM) three pedipalps were prepared after incubation in Karnovsky’s prefixative overnight (see protocol above), in the following way: after dehydration, samples were stained overnight using a 1% iodine solution (in pure ethanol). After washing in pure ethanol, samples were critical point dried (Leica) and subsequently mounted on insect pins using super glue. Scans were performed in an Xradia XCT-200 (Carl Zeiss Microscopy GmbH) using the 20x and 40x objective lens unit with the following scan parameters: 40 kV, 8 W, 200 µA, exposure time 30 sec/frame. Reconstructed image stacks were created using XMReconstructor software (Carl Zeiss Microscopy GmbH). Subsequent segmentation (delineation) of the structures of interest in the male was performed with Amira 5.4.5 (Visualization Science Group, FEI). We reconstructed the neural tissue by comparing MicroCT images with semi-thin sections. The terminology for the description of the neural tissue is according to the neuroglossary established by Richter *et al*.^48^ (2010).
